# Sample entropy characteristics of movement for four foot types based on plantar centre of pressure during stance phase

**DOI:** 10.1186/1475-925X-12-101

**Published:** 2013-10-10

**Authors:** Zhanyong Mei, Guoru Zhao, Kamen Ivanov, Yanwei Guo, Qingsong Zhu, Yongjin Zhou, Lei Wang

**Affiliations:** 1 Shenzhen Institutes of Advanced Technology, The Shenzhen Key Laboratory for Low-cost Healthcare, 1068 Xueyuan Avenue, Shenzhen University Town, Shenzhen 518055, People’s Republic of China; 2 Graduate University of Chinese Academy of Sciences, Beijing 10049, PR China

**Keywords:** Gait, Foot pressure, Velocity, Acceleration, Foot types, Biomechanics

## Abstract

**Background:**

Motion characteristics of CoP (Centre of Pressure, the point of application of the resultant ground reaction force acting on the plate) are useful for foot type characteristics detection. To date, only few studies have investigated the nonlinear characteristics of CoP velocity and acceleration during the stance phase. The aim of this study is to investigate whether CoP regularity is different among four foot types (normal foot, pes valgus, hallux valgus and pes cavus); this might be useful for classification and diagnosis of foot injuries and diseases. To meet this goal, sample entropy, a measure of time-series regularity, was used to quantify the CoP regularity of four foot types.

**Methods:**

One hundred and sixty five subjects that had the same foot type bilaterally (48 subjects with healthy feet, 22 with pes valgus, 47 with hallux valgus, and 48 with pes cavus) were recruited for this study. A Footscan® system was used to collect CoP data when each subject walked at normal and steady speed. The velocity and acceleration in medial-lateral (ML) and anterior-posterior (AP) directions, and resultant velocity and acceleration were derived from CoP. The sample entropy is the negative natural logarithm of the conditional probability that a subseries of length m that matches pointwise within a tolerance r also matches at the next point. This was used to quantify variables of CoP velocity and acceleration of four foot types. The parameters *r* (the tolerance) and *m* (the matching length) for sample entropy calculation have been determined by an optimal method.

**Results:**

It has been found that in order to analyze all CoP parameters of velocity and acceleration during the stance phase of walking gait, for each variable there is a different optimal r value. On the contrary, the value m=4 is optimal for all variables.

Sample entropies of both velocity and acceleration in AP direction were highly correlated with their corresponding resultant variables for r>0.91. The sample entropy of the velocity in AP direction was moderately correlated with the one of the acceleration in the same direction (r≥0.673), as well as with the resultant acceleration (r≥0.660). The sample entropy of resultant velocity was moderately correlated with the one of the acceleration in AP direction, as well as with the resultant acceleration (for the both r≥0.689). Moderate correlations were found between variables for the left foot and their corresponding variables for the right foot.

Sample entropies of AP velocity, resultant velocity, AP acceleration, and resultant acceleration of the right foot as well as AP velocity and resultant velocity of the left foot were, respectively, significantly different among the four foot types.

**Conclusions:**

It can be concluded that the sample entropy of AP velocity (or the resultant velocity) of the left foot, ML velocity, resultant velocity, ML acceleration and resultant acceleration could serve for evaluation of foot types or selection of appropriate footwear.

## Background

Foot problems are often seen among people of all ages over the world. The situation worsens in case of society aging since the incidence of foot diseases grows as age increases [1]. The common foot types are normal foot, pes valgus, pes cavus and hallux valgus. Pes valgus (also known as pes planus, flat foot etc.) is characterized with a collapse of the medial longitudinal arch. Hallux valgus exhibits medial deviation of the first metatarsal. Pes cavus (also known as high arch, cavoid foot etc.) has a higher medial longitudinal arch. Each of these abnormalities leads to a higher incidence of specific complications. For example, people suffering from high arched feet are more likely to develop tibial and femoral stress fractures, and low-arched people are more likely to develop metatarsal injuries [2]; hallux valgus may lead to metatarsalgia, plantar callosities, hammer toe deformities; all these conditions worsen life quality [3,4].

However, in the most countries the available podiatric resources are not enough to solve all foot problems [5]. This leads to a lack of early foot problem recognition. Furthermore, most of the people are not aware of their own foot types [6], and if unsuitable footwear is selected the foot condition will worsen the situation. For these reasons, it is necessary to investigate the characteristics of different foot types to automatically detect foot types.

In gait and posture research, plantar pressure measurement is important to determine the condition of foot and ankle. Such measurement is used in footwear design [7,8], gait identification [9], gait alteration recognition [10], etc. It is also used to investigate the loading characteristics and classification of foot types.

Bavornrit et al. [11] found that, compared with the normal foot, the low arch foot presents an increased contact area beneath the medial midfoot and a decreased peak pressure beneath the lateral forefoot. Pes planus presents significantly more force at the subhallucal area with no difference seen under the other areas [12]. Significant difference between normal foot and pes cavus exists for pressure–time integrals [13]. The peak pressure under first metatarsal and second metatarsal region and the pressure–time integral at the first metatarsal region in hallux valgus group were higher than those in the control group. People with lesser toes deformity exhibited higher pressure–time integral and peak pressure under the 2nd and 3rd metatarsal joints and 2nd toe and 3rd-5th toes compared with people who had normal feet [14]. There are also differences between hallux valgus and normal foot in duration of the forefoot loading and medial-to-lateral transition and in balance pattern [15].

Features obtained from CoP were also used to analyze motion characteristics of different foot types. Ledoux et al. and Hillstrom et al. [12,16] found that the pes planus has lower center of pressure excursion index (CPEI, a measure of CoP concavity in the metatarsal area divided by foot width during stance phase) in comparison with the neutrally aligned foot and cavus foot. The CoP displacement and velocity were investigated for high arch, normal and low arch feet, and the low arch foot exhibited a more lateral COP course [17].

Spatio-temporal variables of plantar pressure were also used to classify the foot types. For example, Bertani et al. [18] used a heuristic optimization (based on Discard-Insert-Exchange optimization method) to choose the most discriminative samples of GRF (ground reaction force) as features for classifying foot types into normal foot and flat foot. Xu et al. [19] proposed a neural network combined with fuzzy logic for classifying feet into normal foot, pes cavus and pes valgus based on four static characteristic indexes: Staheli index (the ratio of the minimum width of midfoot to the maximum width of rearfoot), Chippaux-Smirak index (the ratio of the minimum width of midfoot to maximum width of forefoot), arch index (the ratio of the area of the middle third of the toeless footprint to the entire footprint area) and modified arch index (it is similar to the Arch Index, but it refers the ratio of the sum of the pressures). De Cock et al. [20] selected as features peak pressures, regional impulses and relative regional impulses under hallux, medial and lateral heel and five metatarsal joints; then they used K-mean cluster algorithm to classify pressure patterns into four categories. The K-mean cluster algorithm is a partition-based cluster method; observations are partitioned into k clusters according to the distance between each observation and each centroid.

Until now, linear features are usually extracted from CoP of different foot types. To some degree, they can indicate the biomechanical characteristics of different foot types. However, the linear features which were always extracted from one or several important data points (e.g. maximal force, CPEI) are easily contaminated with noise. Averaging is always used to deal with multiple measurements of each subject; this leads to suppress the time-evolving structure of CoP variability that is regarded as deterministic property [21]. In fact, human gait is a rhythmical oscillation and the movements do not repeat precisely in every gait cycle, thus in every human there is a stride-to-stride variability. Examining this variability allows identifying pathologies of neuromusculoskeletal system.

It is possible for CoP progression patterns of the four foot types to be different from one to another because each of the four foot types has its typical anatomical structure. To investigate the CoP differences of the four foot types, sample entropy has been used in this study. Sample entropy is a complexity measure of time series without counter of self-matches. This was proposed by Richman and Moorman [22]. It is not sensitive to data corrupted by noise [23] and can be used to quantify the stride-to-stride fluctuation of CoP. Sample entropy has been used to analyze CoP data during standing condition [24,25]. To the best of our knowledge, it has not been used to analyze CoP data during the stance phase, especially for the CoP velocity and acceleration of the four foot types. It has been hypothesized that there are differences in sample entropy values for CoP variables among the four foot types. The aim of this study is 1) to investigate whether different foot types could present difference in sample entropy for every CoP variable; 2) to study the relationship between CoP components; 3) to study whether sample entropy of left feet and the one of the right feet have the same statistical characteristics.

The rest of the paper is organized as follows: in Method Section the experimental platform and the way to acquire the data are described; then, sample entropy algorithm is introduced of which two important parameters m and r are determined. Statistical analysis methods are given in Statistical analysis Section. Results from comparison of entropy values of the four foot types are reported in Result Section. Discussion and conclusion are presented in Discussion Section.

## Method

### Experimental data acquisition

**Figure 1:**
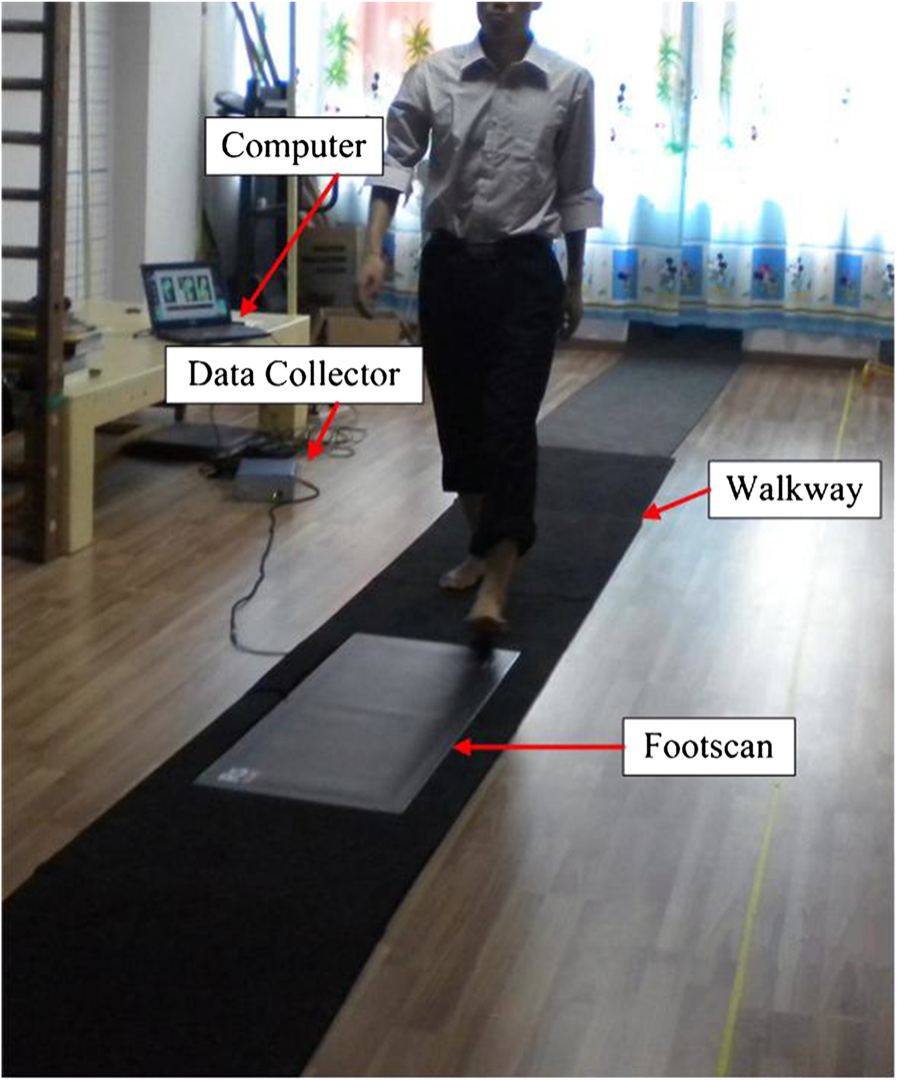
**It consists of Footscan® sensor array, data collector, walkway and computer.** Mid-step protocol was used to acquire plantar pressure data.

A Footscan® system (RSscan International, Olen, Belgium, 1068×418×12 (mm), 1 m, 8192 sensors with density of 2.6 sensors per square centimeter and sample rate of 253 Hz) was used for acquisition of CoP data (including CoP trajectory in ML and AP directions, and the vertical ground reaction force [VGRF]). It was mounted in the middle of an 8 m walkway. The experimental setup is illustrated in Figure 1.

In total, 165 subjects (88 females, 77 males, foot size: 24.4±1.4 cm, weight: 58.7±15.4 kg, height: 162.9±9.5 cm, age: 32.6±14.2 years) were recruited in the study. Among them there were 22 subjects with pes valgus, 48 with pes cavus, 47 with hallux valgus and 48 with normal feet. Subjects who had pes valgus and hallux valgus present together on the same foot were excluded from the study. The both feet of each recruited subject were of the same type.

Each participant was examined by a podiatrist to make sure that he/she is free from other musculoskeletal or neurological diseases. Subjects who had lower extremity injuries within the past six months or underwent foot or ankle surgery, were not included into analysis. The demographic characteristics of all subjects are shown in Table 1. The experimental procedure was approved by the ethics committee of Shenzhen Institutes of Advanced Technology. Each subject read and signed informed consent prior to testing.

**Table 1:** Subjects’ demographic characteristics: allocation of the foot types among subjects, mean age and standard deviation of age, height, weight, and foot size

**Foot Type**	**Age, years Mean (std.dev.)**	**Females/males**	**Height, cm Mean (std.dev.)**	**Weight, kg Mean (std.dev.)**	**Foot size, cm Mean (std.dev.)**
Normal foot	36.5 (12.1)	23/25	163.9 (8.3)	60.4 (11.8)	24.6 (1.4)
Pes cavus	32.1 (12.8)	20/28	166.6 (8.7)	62.4 (16.0)	24.7 (1.5)
Hallux valgus	35.8 (15.3)	37/10	160.5 (6.4)	54.6 (10.3)	23.9 (1.1)
Pes valgus	26.3 (16.3)	8/14	157.8 (14.6)	55.8 (25.0)	24.4 (1.9)
Whole group	32.6 (14.2)	88/77	162.9 (9.5)	58.7 (15.4)	24.4 (1.4)

People tend to spend most of the time in their daily life walking at their normal or comfortable speed. Thus the biomechanical characteristics and those of the plantar pressure for normal walking speed are representative. For this reason plantar pressure data at normal walking speed and a mid-step protocol was used (i.e. the subject was required to make at least three steps before and after contacting the Footscan [26,27]). A trial was considered valid when the following criteria were met: (1) Subjects were instructed to look forward and not to look at the pressure plate and the walkway. (2) Each subject has not suddenly changed his/her gait before and after accessing the plate i.e. there were no changes in the step length, the cadence, etc. (3) Plantar contact area was confined into the measurement area. (4) For each subject qualified plantar data was acquired six times for each foot.

### Sample entropy

**Figure 2:**
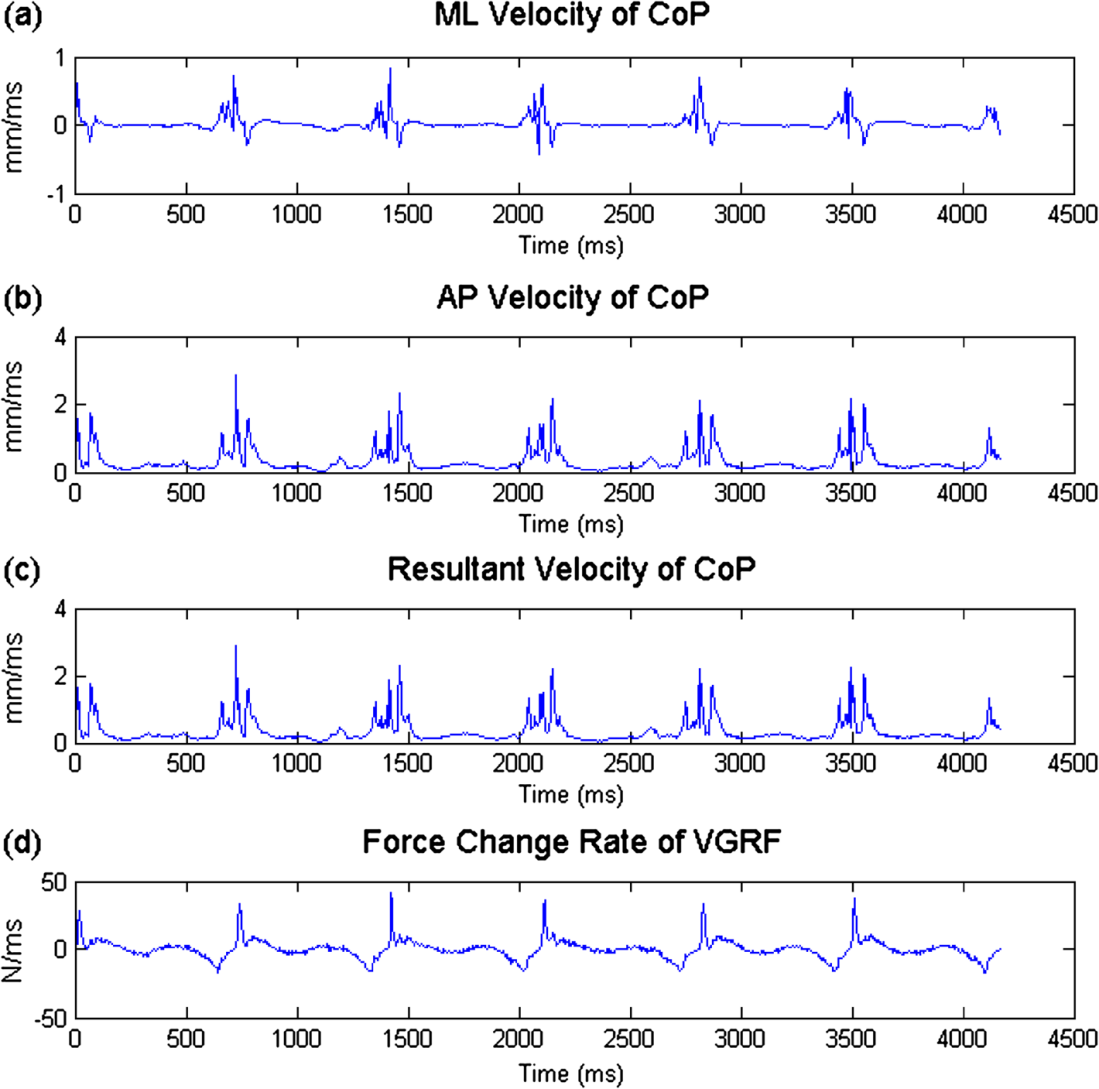
Illustration of ML velocity (a) and AP velocity (b), resultant velocity, (c), and force change rate (d) of one subject with hallux valgus.

**Figure 3:**
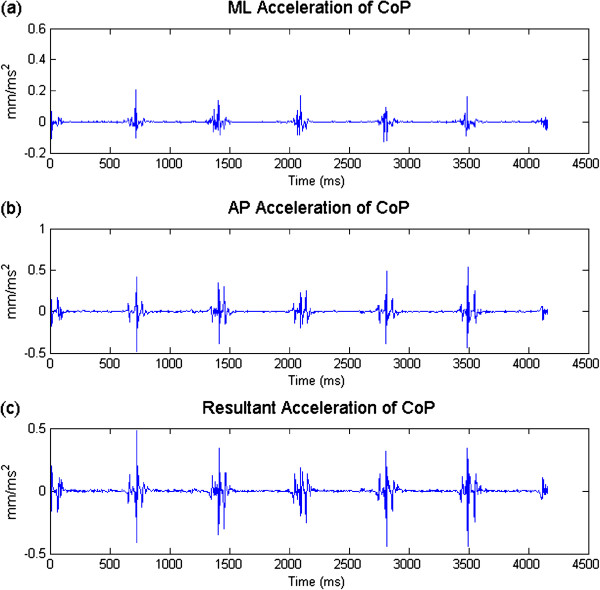
Illustration of ML acceleration (a) and AP accelerations (b), and resultant acceleration (c) of one subject with hallux valgus.

In this study, CoP velocity and acceleration in ML and AP directions, CoP resultant velocity and acceleration, and force change rate, which derived from CoP trajectory and VGRF, were quantified using sample entropy. The velocity and acceleration are defined as follows:

(1) $$V_{ML_{i}}=\frac{X_{i+1}-X_{i}}{\Delta t}$$

(2) $$V_{AP_{i}}=\frac{Y_{i+1}-Y_{i}}{\Delta t}$$

(3) $$V_{R_{i}}=\frac{\sqrt{{\left(X_{i+1}-X_{i}\right)}^{2}+{\left(Y_{i+1}-Y_{i}\right)}^{2}}}{\Delta t}$$

(4) $$V_{F_{i}}=\frac{F_{i+1}-F_{i}}{\Delta t}$$

(5) $$A_{ML_{i}}=\frac{V_{ML_{i}}{}_{{}_{+1}}-V_{ML_{i}}}{\Delta t}$$

(6) $$A_{AP_{i}}=\frac{V_{AP_{i+1}}-V_{AP_{i}}}{\Delta t}$$

(7) $$A_{R_{i}}=\frac{V_{R_{i+1}}-V_{R_{i}}}{\Delta t}$$

where *X*_*i*_, *Y*_*i*_ and *F*_*i*_ are the coordinates of CoP in ML and AP directions, and the force at a given time *i*, respectively. *Δt* represents the sampling interval. $V_{ML_{i}}$, $V_{AP_{i}}$, $V_{R_{i}}$ and $V_{F_{i}}$ are the velocities in ML and AP directions, the resultant velocity, and the force change rate, respectively. $A_{ML_{i}}$, $A_{AP_{i}}$ and $A_{R_{i}}$ represent the accelerations in ML and AP directions and the resultant acceleration in both directions. CoP data of all trials for the left foot and right foot of each subject were concatenated as whole time series. In Figure 2 and Figure 3 the time series for all variables related to velocity and acceleration are illustrated. The variables CoP velocity and acceleration for left foot and right foot are listed in Table 2. Before calculating the sample entropy, each time series was normalized to unit variance by dividing it by its respective standard deviation.

**Table 2:** The abbreviated variables

	**The abbreviated variables**	**The meaning of CoP variables**
Left foot	L_ML_V	Medial-lateral velocity of CoP for left foot
	L_AP_V	Anterior-posterior velocity of CoP for left foot
	L_Res_V	Resultant velocity of CoP for left foot
	L_F_V	Force change rate of vertical ground reaction force for left foot
	L_ML_A	Medial-lateral acceleration of CoP for left foot
	L_AP_A	Anterior-posterior acceleration of CoP for left foot
	L_Res_A	Resultant acceleration of CoP for left foot
Right foot	R_ML_V	Medial-lateral velocity of CoP for right foot
	R_AP_V	Anterior-posterior velocity of CoP for right foot
	R_Res_V	Resultant velocity of CoP for right foot
	R_F_V	Force change rate of ground vertical reaction force for right foot
	R_ML_A	Medial-lateral acceleration of CoP for right foot
	R_AP_A	Anterior-posterior acceleration of CoP for right foot
	R_Res_A	Resultant acceleration of CoP for right foot

Sample entropy is the negative natural logarithm of the conditional probability that a subseries of length m that matches pointwise within a tolerance r will also match at the next point [28]. Moreover, sample entropy derived from approximate entropy without counting self-matches. Lower sample entropy value indicates more self-similarity in the time series. In addition, for eliminating self-matches, sample entropy algorithm is simpler than approximate entropy algorithm, and its execution time is approximately the half of the one that is required by the approximate entropy algorithm. Sample entropy is largely independent of record length and demonstrates relative consistency under circumstances where approximate entropy does not [22,29].

The algorithm to calculate sample entropy (SamEn) is as follows [28]:

Given a time sequence data *x*_*1*_, *x*_*2*_, *…*, *x*_*n*_, n is the total number of data points. First, it is necessary to construct a subseries (template vector) of length m: *X*_*i*_=[*x*_*i*_*, x*_*i+1*_*,…, x*_*i+m-1*_], where i=1, 2, …, n-m+1; m represents embedding dimension.

After this, the probability that any of the vectors will be similar with *Xi* is calculated:

(8) $$C_{i}=\frac{n_{i}\left(m,r\right)}{N-m+1}$$

where *n*_*i*_*(m, r)* is the number of vectors *X*_*j*_ that are similar to *X*_*i*_ with the following constrain:

(9) $$d\left(X_{i},X_{j}\right)\leq r$$

where *d(X*_*i*_*, X*_*j*_*)* is defined as the maximal absolute difference between vectors *X*_i_ and *Y*_j_ in their respective scalar components; r specifies the filter level (tolerance). If the distance between *X*_*i*_ and *X*_*j*_ is less than r, the counter of vectors which are similar to *X*_*i*_ will increase by one. In the above counter of similar matches, *j=i* cases are not included to avoid self-match counting.

Then, calculate the average probability:

(10) $$\Phi \left(m,r\right)=\frac{1}{N-m+1}\sum_{i=1}^{N-m+1}C_{i}\left(m,r\right)$$

Similar process is repeated for an embedded dimension m+1 to calculate *Φ*(*m* + 1, *r*).

Sample entropy is given as

(11) $$\text{SamEn}\left(X_{N},m,r\right)=-\ln\frac{\Phi \left(m+1,r\right)}{\Phi \left(m,r\right)}$$

To calculate the sample entropy for each of the CoP measurement variables, the parameters *m* and *r* should be determined. As CoP data were acquired during the stance phase, *m* and *r* may be different from the recommended values (*m* can take value 1 or 2, *r* can take values between 0.1 and 0.25 [30]). The method proposed in [31] and [32] was used to determine the values of *m* and *r*.

If the number of matches of length m and m+1 increase, the accuracy and confidence of sample entropy estimate will increase. Small m values and large r values result into an increased number of matches. However, as r increases, the probability of match will tend to 1, thus the quantified sample estimate will lose the discriminative ability; as m decreases, underlying physical process may be obscured [32]. Therefore, m values and r values should be rationally chosen for reliable estimation of sample entropy.

**Figure 4:**
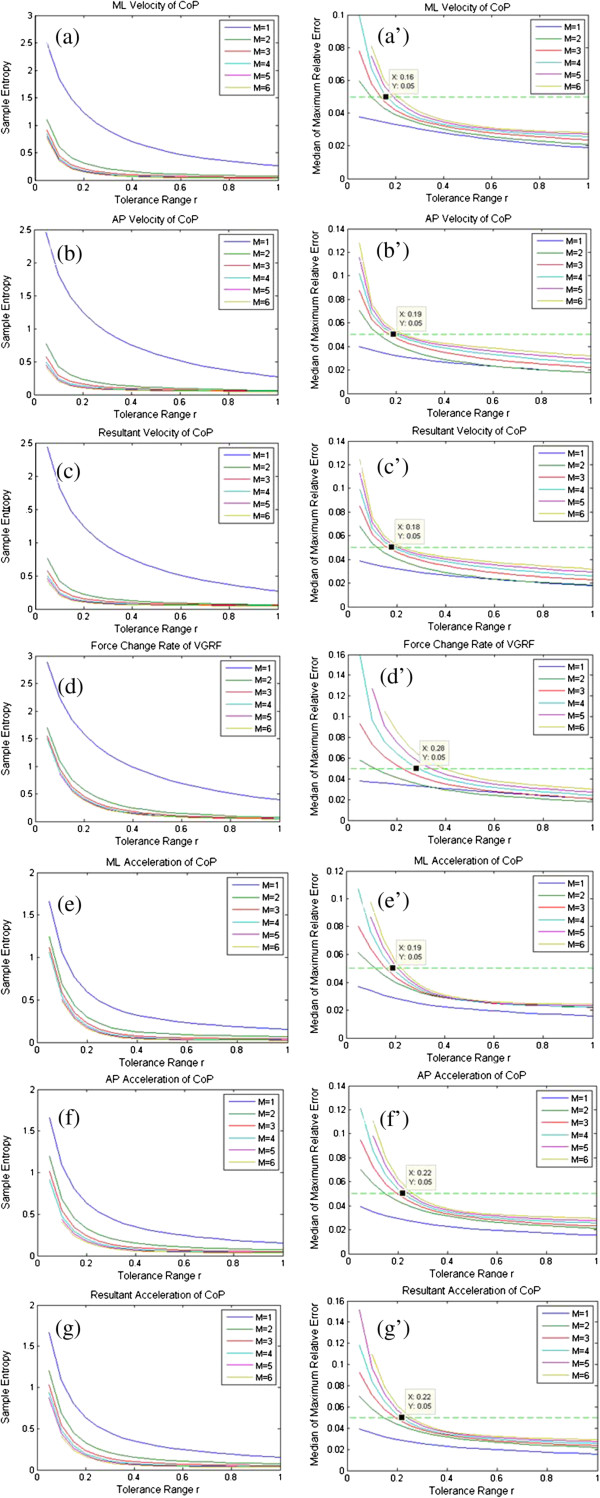
**Optimal selections of parameters m and r.** In figures **(a)**-**(g)**, median sample entropy is calculated over all time series of each variable. When m≥4, the curves converge. In figures **(a’)**-**(g’)**, medians of maximum relative error that correspond to different m and r values are illustrated. Each r value was determined under the condition that the median of the maximum relative error is no more than 0.05.

To find rational *m* values, first the sample entropy of each time-series with combination of m from 1 to 6 with step 1, and r from 0.1 to 1 with step 0.05 has been calculated; then median sample entropy has been obtained for given m and r. The median sample entropy for each CoP variable is illustrated in Figure 4 (a)-(g). It can be seen that the median sample entropy of all variables converges when m≥4 for almost all r values.

To estimate the appropriate *m* and *r* values, conditional probability has been calculated:

(12) $$\mathrm{CP}\left(m,r\right)=\frac{A\left(r\right)}{B\left(r\right)}$$

where *A(r)* and *B(r)* are, respectively, the number of matches of length m+1 and m within tolerance r. The variance of CP can be estimated as:

(13) $$\sigma_{\mathrm{CP}}^{2}=\frac{\mathrm{CP}\left(1-\text{CP}\right)}{B}+\frac{1}{B^{2}}\left[K_{A}-K_{B}{\left(\text{CP}\right)}^{2}\right]$$

where *K*_*A*_ and *K*_*B*_ are, respectively, the number of pairs of matching templates of length m+1 and m that overlap within tolerance r. The r values are determined by minimizing the quantity:

(14) $$Q\left(m,r\right)=\max\left(\frac{\sigma_{\text{CP}}\left(m,r\right)}{\text{CP}\left(m,r\right)},\frac{\sigma_{\text{CP}}\left(m,r\right)}{-\log\left(\text{CP}\left(m,r\right)\right)\text{CP}\left(m,r\right)}\right)$$

where *Q(m, r)* is the maximum relative error of SampEn and the CP estimate.

The metric simultaneously penalizes CP near 0 and 1 and it is a tradeoff between accuracy and discriminative capability. The maximal relative error is set less than 0.05; this value corresponds to 95% confidence interval, which is 10% sample entropy estimation.

The median between the maximum relative error for m≥4 and all r values for each CoP variable has been calculated. In Figure 4 (a’)-(g’), the medians of maximum relative error for m=4, 5, 6 are illustrated. Under the condition that the median of maximum relative error is set to 0.05, in order to reach the best discriminative ability of sample entropy, the values have been set as follows: for m a value equal to 4 has been chosen. On the contrary the r values of all variables for the left foot should be: ML velocity (L_ML_V) 0.16, AP velocity (L_AP_V) 0.19, resultant velocity (L_Res_V) 0.18, force change rate (L_F_V) 0.28, ML acceleration (L_ML_A) 0.19, AP acceleration (L_AP_A) 0.22, resultant acceleration (L_Res_A) 0.22; the corresponding variables for the right foot should have values of 0.17, 0.18, 0.17, 0.29, 0.18, 0.21and 0.20, respectively.

### Statistical analysis

To investigate the relationship between variables for left foot or right foot, Spearman Correlation analysis was performed because some variables did not follow normal distribution. If the coefficient |r|≥0.75, the two variables highly correlate; if 0.25≤|r|<0.75, the two variables moderately correlate; if |r|<0.25, the two variables weakly correlate [33]. The sample entropy of each variable was log-transformed to achieve normal distribution and equality of variance. Because of the inequality of variables R_ML_V, R_AP_V, L_ML_V and L_ML_A, Kruskal-Wallis test was applied to investigate whether sample entropy is statistically different for each of the three variables among the four foot types. Analysis of variance was performed with pairwise comparisons with Bonferroni adjustment for the rest variables (L_AP_V, L_Res_V, L_F_V, L_AP_A, L_Res_A, R_Res_V, R_F_V, R_ML_A, R_ AP_A, and R_Res_A).

With regard to the corresponding variables of left foot and right foot, if there is no high correlation between them and analysis of variance or Kruskal-Wallis test shows that there is a statistical difference between them, then data from the left foot and the right foot should be collected and processed separately. On the other hand, with regard to the CoP variables of the same-side foot, if there is a high correlation between variables and analysis of variance or Kruskal-Wallis test shows that they have similar statistical characteristics, then one of the variables cannot be used for classification of foot types or evaluation of foot function. All statistics were calculated with SPSS 17.0 and P<0.05 was taken as significant level.

## Result

### Relationships between variables

In Tables 3, 4 and 5, Spearman correlation coefficients and p-values are listed for all paired parameters. For left foot, AP velocity (L_AP_V) is highly correlated with the resultant velocity (L_Res_V) (r=0.971, p=0.000) and AP acceleration (L_AP_A) is also highly correlated with the resultant acceleration (L_Res_A) (r=0.985, p=0.000). With regard to the correlations between velocity parameters and acceleration parameters, there is a moderate correlation between the ML velocity (L_ML_V) and ML acceleration (L_ML_A) (r=0.662, p=0.000); AP velocity (L_AP_V) and AP acceleration (L_AP_A) are moderately correlated (r=0.723, p=0.000); there was a moderate correlation between AP velocity (L_AP_V) and resultant acceleration (L_Res_A) (r=0.714, p=0.000), between resultant velocity (L_Res_V) and AP acceleration (L_AP_A) (r=0.742, p=0.000), between resultant velocity (L_Res_V) and resultant acceleration (L_Res_A) (r=0.746, p=0.000). There were weak correlations between other paired parameters. The correlations between variables for the right foot were similar with those for the left foot.

**Table 3:** Spearman correlation coefficients for sample entropy estimate between variables of the left foot

		**L_ML_V**	**L_AP_V**	**L_Res_V**	**L_F_V**	**L_ML_A**	**L_AP_A**	**L_Res_A**
L_ML_V	r	1.000	.278^**^	.271^**^	.191^*^	.662^**^	.192	.196
	p-value		.000	.000	.015	.000	.014	.012
L_AP_V	r	.278^**^	1.000	.971^**^	.259^**^	.335^**^	.723^**^	.714^**^
	p-value	.000		.000	.001	.000	.000	.000
L_Res_V	r	.271^**^	.971^**^	1.000	.246^**^	.348^**^	.742^**^	.746^**^
	p-value	.000	.000		.002	.000	.000	.000
L_F_V	r	.191^*^	.259^**^	.246^**^	1.000	.243^**^	.329^**^	.324^**^
	p-value	.015	.001	.002		.002	.000	.000
L_ML_A	r	.662^**^	.335^**^	.348^**^	.243^**^	1.000	.394^**^	.427^**^
	p-value	.000	.000	.000	.002		.000	.000
L_AP_A	r	.192^*^	.723^**^	.742^**^	.329^**^	.394^**^	1.000	.985^**^
	p-value	.014	.000	.000	.000	.000		.000
L_Res_A	r	.196^*^	.714^*^	.746^*^	.324^**^	.427^**^	.985^**^	1.000
	p-value	.012	.000	.000	.000	.000	.000	

^*^Correlation is significant at the 0.05 level (2-tailed).

^**^Correlation is significant at the 0.01 level (2-tailed).

**Table 4:** Spearman correlation coefficients for sample entropy estimate between variables of the right foot

		**R_ML_V**	**R_AP_V**	**R_Res_V**	**R_F_V**	**R_ML_A**	**R_AP_A**	**R_Res_A**
R_ML_V	r	1.000	.255^**^	.302^**^	.254^**^	.697^**^	.187^*^	.243^**^
	p-value		.001	.000	.001	.000	.017	.002
R_AP_V	r	.255^**^	1.000	.963^**^	.314^**^	.229^**^	.673^**^	.660^**^
	p-value	.001		.000	.000	.003	.000	.000
R_Res_V	r	.302^**^	.963^**^	1.000	.265^**^	.279^**^	.669^**^	.681^**^
	p-value	.000	.000		.001	.000	.000	.000
R_F_V	r	.254^**^	.314^**^	.265^**^	1.000	.340^**^	.408^**^	.427^**^
	p-value	.001	.000	.001		.000	.000	.000
R_ML_A	r	.697^**^	.229^**^	.279^**^	.340^**^	1.000	.422^**^	.511^**^
	p-value	.000	.003	.000	.000		.000	.000
R_AP_A	r	.187^*^	.673^**^	.669^**^	.408^**^	.422^**^	1.000	.975^**^
	p-value	.017	.000	.000	.000	.000		.000
R_Res_A	r	.243**	.660**	.681**	.427**	.511**	.975**	1.000
	p-value	.002	.000	.000	.000	.000	.000	

^*^Correlation is significant at the 0.05 level (2-tailed).

^**^Correlation is significant at the 0.01 level (2-tailed).

**Table 5:** Spearman correlation coefficients for sample entropy estimate between variables of left foot and right foot

		**R_ML_V**	**R_AP_V**	**R_Res_V**	**R_F_V**	**R_ML_A**	**R_AP_A**	**R_Res_A**
L_ML_V	r	.637^**^	.058	.067	.204^**^	.393^**^	.056	.089
	p-value	.000	.458	.399	.009	.000	.476	.256
L_AP_V	r	.214^**^	.586^**^	.564^**^	.329^**^	.246^**^	.443^**^	.464^**^
	p-value	.006	.000	.000	.000	.002	.000	.000
L_Res_V	r	.192^*^	.583^**^	.574^**^	.328^**^	.236^**^	.454^**^	.481^**^
	p-value	.014	.000	.000	.000	.002	.000	.000
L_F_V	r	.163^*^	.227^**^	.193^*^	.738^**^	.320^**^	.330^**^	.348^**^
	p-value	.038	.004	.014	.000	.000	.000	.000
L_ML_A	r	.389^**^	-.008	-.007	.292	.453^**^	.182^*^	.223^**^
	p-value	.000	.920	.934	.000	.000	.020	.004
L_AP_A	r	.097	.398^**^	.405^**^	.396^**^	.260^**^	.601^**^	.611^**^
	p-value	.217	.000	.000	.000	.001	.000	.000
L_Res_A	r	.097	.381^**^	.393^**^	.387^**^	.267^**^	.587^**^	.601^**^
	p-value	.217	.000	.000	.000	.001	.000	.000

^*^Correlation is significant at the 0.05 level (2-tailed).

^**^Correlation is significant at the 0.01 level (2-tailed).

Moderate correlations were found between variables for left foot and corresponding variables for the right foot. With regard to the variables, AP velocity of the left foot (L_AP_V) was moderately correlated with the resultant velocity of the right foot (R_Res_V) (r=0.564, p=0.000); resultant velocity of the left foot (L_Res_V) was also moderately correlated with AP velocity of the right foot (R_AP_V) (r=0.583, p=0.000); AP acceleration of the left foot (L_AP_A) was moderately correlated with resultant acceleration of the right foot (R_Res_A) (r=0.611, p=0.000); resultant acceleration of the left foot (L_Res_A) was also moderately correlated with AP acceleration of the right foot (R_AP_A) (r=0.587, p=0.000).

### Comparisons of sample entropy for velocity and acceleration variables

The result of Kruskal-Wallis test for the variables is given in Table 6; for the left foot, there are no significant differences for measurements of ML velocity and ML acceleration (p=0.510 and p=0.466, respectively). For the right foot, there is no difference in sample entropy of ML velocity among the four groups; significant difference was found in the comparison of AP velocity among the four groups.

**Table 6:** Kruskal-Wallis test for variables of velocity and acceleration for left foot and right foot

		**Mean rank**				***χ***^**2**^	**p-value**
		**Normal Foot**	**Pes Valgus**	**Hallux Valgus**	**Pes Cavus**		
Left foot	L_ML_V	75.38	76.10	88.28	85.13	2.312	.510
	L_ML_A	75.21	75.05	86.09	87.96	2.550	.466
Right foot	R_ML_V	71.10	74.57	94.36	84.09	6.394	.094
	R_AP_V	85.17	96.43	95.83	58.49	17.876	.000

Mean and standard deviations, and pairwise comparisons for variables are listed in Table 7. For measurements of left foot, sample entropy of AP velocity (L_ML_V) and resultant velocity (L_Res_V) of pes cavus were significantly different from those of the other three foot types; there was no significant difference in pairwise comparisons of sample entropy of force change rate (L_F_V), AP acceleration (L_AP_A) and resultant acceleration (L_Res_A) between any two foot types.

**Table 7:** Mean and standard deviations, and pairwise comparisons of sample entropy between normal foot, pes cavus, pes valgus, and hallux valgus

		**Mean(STD)**			
		**Normal Foot**	**Pes Valgus**	**Hallux Valgus**	**Pes Cavus**
Left foot	L_AP_V	−2.000 (.358)^d^	−1.890 (.358)^d^	−1.998(.324)^d^	−2.180(.303)^d^
	L_Res_V	−1.970(.363)^d^	−1.866(.324)^d^	−1.959(.330)^d^	−2.151(.310)^d^
	L_F_V	−1.359(.267)	−1.426(.352)	−1.332(.310)	−1.429(.317)
	L_AP_A	−1.914 (.790)	−2.050 (.941)	−1.890(.710)	−2.104(.635)
	L_Res_A	−1.917 (.774)	−2.066 (.886)	−1.873 (.703)	−2.104(.634)
Right foot	R_Res_V	−1.968(.290)^d^	−1.960(.418)^d^	−1.878 (.341)^d^	−2.149(.260)^d^
	R_F_V	−1.439 (.280)	−1.452 (.289)	−1.408 (.306)	−1.504(.324)
	R_ML_A	−1.750(.621)	−1.917(.686)	−1.650(.729)	−1.652(.816)
	R_AP_A	−1.825(.652)^bd^	−2.180(.889)^a^	−1.826(.681)^d^	−2.149(.621)^ac^
	R_Res_A	−1.781(.650)^bd^	−2.159(.832)^ac^	−1.790(.653)^bd^	−2.106(.639)^ac^

STD: standard deviation.

^a, b, c, d^: significantly different from normal foot (a), pes valgus (b), hallux valgus (c) and pes cavus (d), respectively.

**Figure 5:**
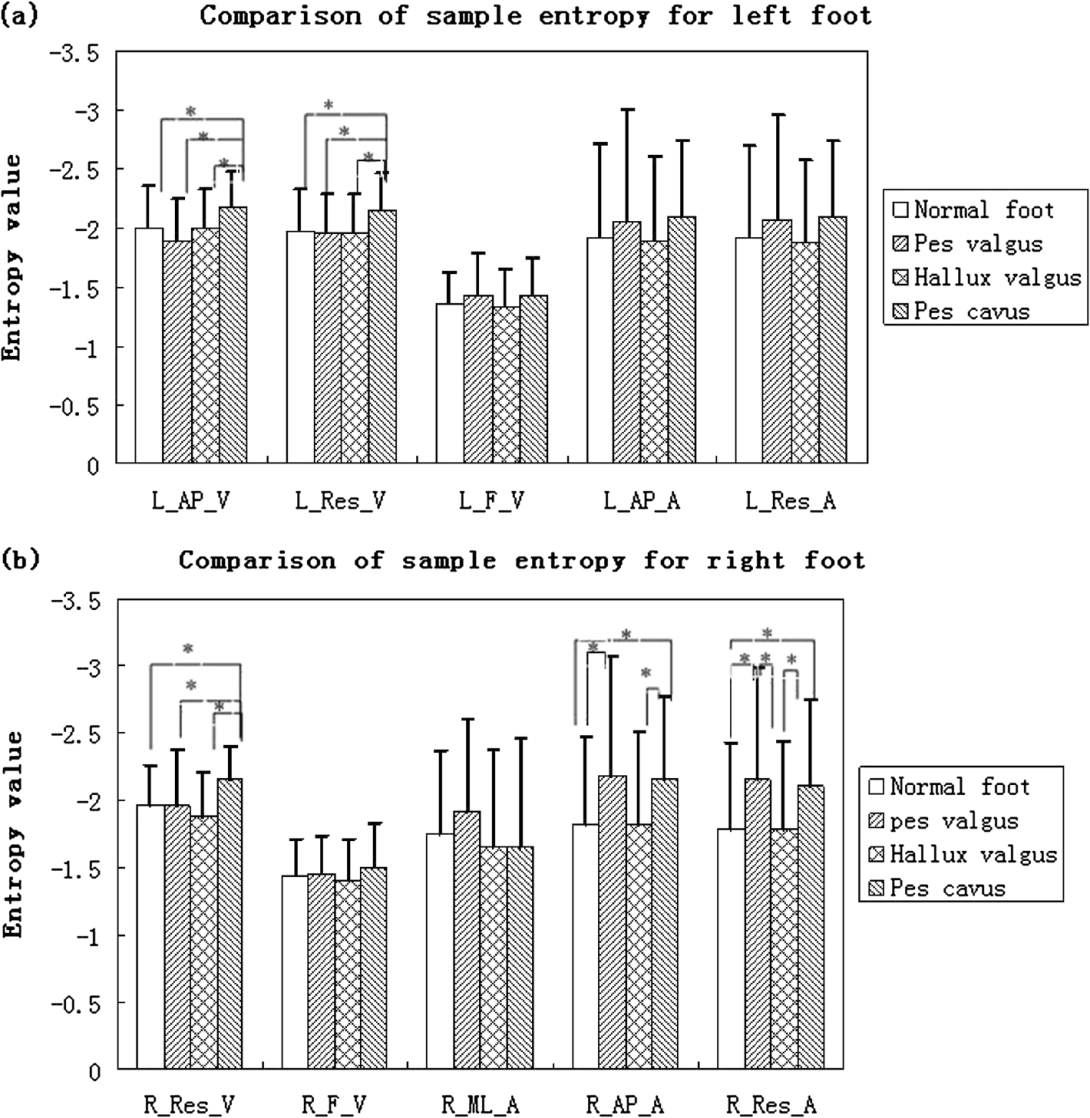
**Multiple intercomparisons of means and standard deviations of sample entropy between normal foot, pes cavus, pes valgus and hallux valgus for ML and AP velocity and acceleration, resultant velocity and acceleration and force change rate.** Comparison for variables of left foot **(a)** and right foot **(b)**.

For measurements of the right foot, sample entropy of AP velocity (R_AP_V) of pes cavus was significantly different from the ones of normal foot, pes valgus and hallux valgus (p=0.006, 0.025, 0.000, respectively). Sample entropy of AP acceleration (R_AP_A) of normal foot was significantly different from the ones of pes valgus and pes cavus (p=0.050, 0.022, respectively). Sample entropy of AP acceleration (R_AP_A) of hallux valgus was significantly different from the one of pes cavus (p=0.024). Sample entropies of the resultant accelerations (R_Res_A) of normal foot and hallux valgus were significantly different from the ones of the resultant accelerations of pes valgus and pes cavus; in particular: for the pair “normal foot - pes valgus” p=0.029; for the pair “normal foot-pes cavus” p=0.020; for pair “hallux valgus-pes valgus” p=0.39; for the pair “hallux vagus-pes cavus” p=0.24. No differences were found in the pairwise comparisons of sample entropy of force change rate (R_F_V) and the one of ML acceleration (R_ML_A) among the four foot types. Pairwise comparisons for above variables for left foot and right foot are shown in Figure 5 (a) and Figure 5 (b), respectively.

## Discussion

This study was conducted to investigate whether there is a difference in sample entropy of each CoP variable among four foot types. Optimal values for m and r (which should be determined before calculation of the sample entropy) for every variable were found and they were different from those that were used in the analysis of the CoP sway data (m=3, r=0.3 [24]). Statistical differences were found for sample entropy of CoP velocity and acceleration.

### Foot structure and function of different foot types result in different CoP patterns

Above-mentioned statistical characteristics are based on the biomechanics of the different foot types during the stance phase. During this phase, the roll-off process of foot and ankle is accompanied with complex movements including dorsiflexion/plantarflexion on frontal plane, enversion/inversion on transverse plane and adduction/abduction on sagittal plane. Abnormity of foot anatomical structure (e.g. pes cavus, pes valgus, hallux valgus) will exhibit different patterns of the complex movement, and this will result into difference in CoP displacement, velocity and acceleration among the four foot types. For example, the forefoot of the pes planus was less adducted at toe-off in the transverse plane compared with normal foot [34]; at the ankle, the high-arched athletes exhibited significantly smaller peak eversion angles in walking compared to the low-arched athletes [35]. Therefore, complexity of CoP velocity and acceleration could indicate the functional difference of the foot and ankle among the four foot types.

In this study it has been found that sample entropy of ML acceleration and resultant acceleration were different between normal foot and pes valgus. Ledoux et al. [36] found that there is no difference in the acceleration at heel strike between normal foot (neutrally aligned foot) and pes valgus; however, in our study it has been found that sample entropy of AP and resultant acceleration were different between those of the two foot types. This may indicate that, compared with some of the linear methods, sample entropy has certain advantages in providing information about condition of foot and ankle.

### Similarities in sample entropy between velocity/acceleration variables

Spearman correlation indicated that two pairs of variables (AP velocity vs. resultant velocity, AP acceleration vs. resultant acceleration) of the left foot were highly correlated (p>0.96 for the both). Sample entropies for AP velocity and resultant velocity of the left foot exhibited similar statistical characteristics: pes cavus was different from any of the other three foot types. There was no statistical difference for sample entropy of AP acceleration (L_AP_A) and resultant acceleration (L_Res_A) among the four foot types. For the right foot, AP velocity (R_AP_V) and resultant velocity (R_Res_V) also demonstrated similar statistical characteristics. Sample entropies of AP acceleration (R_AP_A) and resultant acceleration (R_Res_A) were also similar with the only exception that the sample entropy of resultant acceleration (R_Res_A) for pes valgus and hallux valgus were different.

In conclusion, sample entropy for AP velocity and resultant velocity, AP acceleration and resultant acceleration had the same statistical characteristics. Actually, CoP displacement in AP direction is larger than the one in ML direction during the rollover process, so at the same time, velocity and acceleration in AP direction are larger than those in ML direction. In other words, ML velocity (and ML acceleration) components almost had no effect on the resultant velocity (and resultant acceleration). The above result seems similar with the one observed by De Cock et al. [17] that the curve of the resultant velocity resembles the curve of velocity in AP direction.

### Differences in sample entropy between right and left foot

There was no significant difference for sample entropy of AP acceleration (L_AP_A) and resultant acceleration (L_Res_A) of the left foot among the four foot types. However, for the right foot, statistically significant differences were found for pairwise comparisons of sample entropy of AP acceleration. It seems that CoP of the right foot can provide more discriminative information than the one of the left foot. The possible reason is that there is a functional asymmetry between left foot and right foot (the left foot is responsible for supporting the body while the right foot is responsible for propulsion during walking [37]). Since the right foot is in charge of propulsion, its sample entropy may exhibit more abnormalities of structure and function among the foot types. Considering the difference between statistical characteristics of the left foot and the right foot, data sets of left and right foot should be collected independently for sample entropy analysis of CoP velocity and acceleration.

### Limitations of the study

In this study, each subject walked many times in order to collect enough plantar pressure information to perform analysis. After this, the CoP time series of the different measurements of each subject were concatenated. If in-shoe plantar pressure device was used, it reduces the number of trails for each subject, and it is also of benefit for the subsequent data processing. In this study only four foot types have been explored. However, each foot type could be divided into sub-groups according to aetiology or symptom, and the pattern of plantar pressure of one sub-group may differ from those of the other sub-groups of the same foot type. For example, plantar pressures of idiopathic and neurogenic pes cavus are different [13], as well as hallux valgus with pain is different than the one without pain [15]. Merging all subgroups of one foot type into a common group may not lead to significant difference when comparing normal foot with a foot from another type. In future studies, more sub-groups will be considered. As a limitation of our method appears the dependence of the sample entropy on the sampling rate. It results from the fact that at higher sampling rates the self-match counter value will be higher. The optimal sampling rate should be determined for each particular system.

## Conclusion

The main object of this study is to investigate the difference of four foot types using sample entropy for quantification of the CoP velocity and acceleration during the stance phase. From the present study the following conclusions can be drawn:

1. In order to analyse CoP data during the stance phase, values of m and r different from the recommended should be used.

2. The statistical characteristics of the corresponding variables differ between the left and the right foot. For this reason CoP data for the left foot and the right foot should be collected and processed separately; in this way the study of CoP non-linear characteristics of different foot types is facilitated.

3. Statistical difference was found for the following variables: sample entropies of AP velocity (R_AP_V), resultant velocity (R_Res_V), resultant acceleration (R_Res_A) of the right foot, sample entropies of AP velocity (L_AP_V) and resultant velocity (L_Res_V).

The results can be potentially used for evaluation of foot function, automatic classification of the foot type, or selection of footwear.

## Competing interests

The authors declare that they have no competing interests.

## Authors’ contributions

ZYM was responsible for the algorithm implementation, statistical analysis and data acquisition. GRZ participated in experimental design and data analysis. KI contributed to data acquisition and the article revision. YWG and QSZ participated in data acquisition and data analysis. YJZ contributed to prepare introduction. LW provided the experimental infrastructure and contributed to the discussion of results. All authors have read and approved the final manuscript.
